# A case of multidrug-resistant intractable pylephlebitis and intra-abdominal abscess due to perforated appendicitis successfully treated with open abdominal management

**DOI:** 10.1186/s40792-024-01882-1

**Published:** 2024-04-12

**Authors:** Yu Norimatsu, Nobuyuki Takemura, Kaoru Yoshikawa, Kyoji Ito, Fuyuki Inagaki, Fuminori Mihara, Kazuhiko Yamada, Norihiro Kokudo

**Affiliations:** Hepato-Biliary-Pancreatic Surgery Division, Department of Surgery, National Center for Global Health and Medicine, 1-21-1 Toyama, Shinjuku-ku, Tokyo, 162-8655 Japan

**Keywords:** Pylephlebitis, Acute severe appendicitis, Open abdominal management, Multidrug-resistant *Bacteroides fragilis*

## Abstract

**Background:**

Pylephlebitis, a rare and lethal form of portal venous septic thrombophlebitis, often arises from infections in regions drained by the portal vein. Herein, we report a case of peritonitis with portal vein thrombosis due to acute severe appendicitis, managed with intensive intraperitoneal drainage via open abdominal management (OAM).

**Case presentation:**

A 19-year-old male with severe appendicitis, liver abscesses, and portal vein thrombosis developed septic shock and multi-organ failure. After emergency interventions, the patient was admitted to the intensive care unit. Antibiotic treatment based on cultures revealing multidrug-resistant *Escherichia coli* and *Bacteroides fragilis* and anticoagulation therapy (using heparin and edoxaban) was initiated. Despite continuous antibiotic therapy, the laboratory results consistently showed elevated levels of inflammatory markers. On the 13th day, open abdominal irrigation was performed for infection control. Extensive intestinal edema precluded wound closure, necessitating open-abdominal management in the intensive care unit. Anticoagulation therapy was continued, and intra-abdominal washouts were performed every 5 days. On the 34th day, wound closure was achieved using the anterior rectus abdominis sheath turnover method. The patient recovered successfully and was discharged on the 81st day.

**Conclusions:**

Alongside appropriate antibiotic selection, early surgical drainage and OAM are invaluable. This case underscores the potential of anticoagulation therapy in facilitating safe surgical procedures.

## Background

Pylephlebitis and septic thrombophlebitis of the portal vein and superior mesenteric veins (SMV) are very uncommon and usually develop secondary to infection in regions drained by the portal system or in structures contiguous with the portal vein [1–3]. Despite major advances in antibiotic therapy, mortality rates remain high, ranging from 11 to 32% [4]. Moreover, although modern imaging techniques provide supportive diagnostic evidence, the clinical diagnosis is sometimes difficult and delayed because of nonspecific symptoms. In this report, we present a case of severe peritonitis accompanied by refractory to conservative treatments by portal vein thrombosis caused by acute severe appendicitis; intensive co-administration of intraperitoneal drainage based on open abdominal management (OAM) was successful in saving the patient.

## Case presentation

**Fig. 1:**
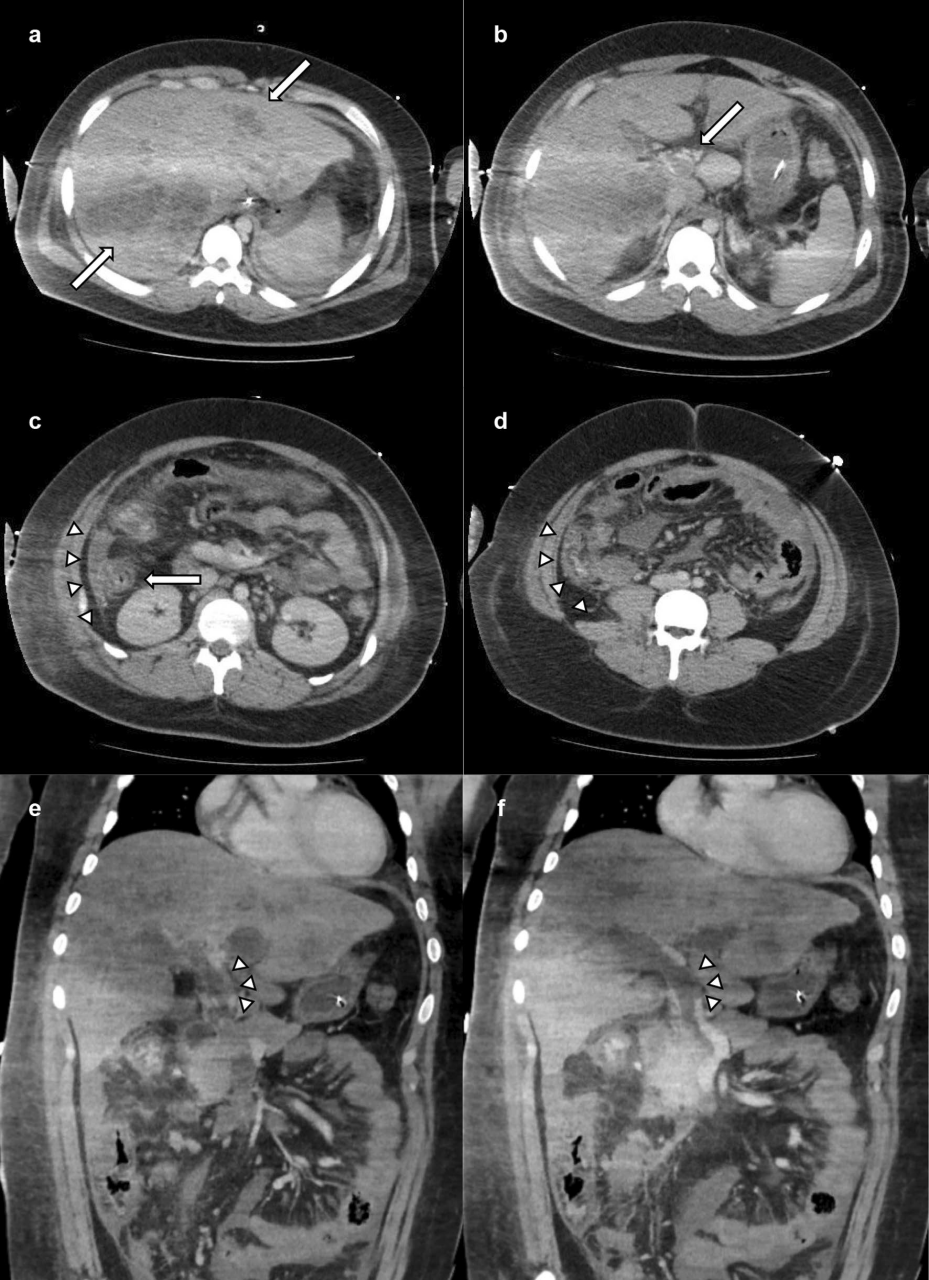
Contrast-enhanced CT at admission revealed **a** multiple bilobular ring-enhanced lesions in the liver (arrows), **b** a low-density area in the portal vein (arrow), **c** an enlarged appendix (arrow), and inflectional ascites in the right paracolic gutter (arrowheads), **e** and **f** a low-density area in the portal vein of the sagittal sections (arrowheads). CT: computed tomography

A 19-year-old male patient presented to the emergency department with a sudden loss of consciousness following a 3-week history of persistent vomiting and diarrhea. This patient had no significant medical history or comorbidities but faced social issues as an international student from China without health insurance. Upon admission, the patient was severely obese (height, 176 cm; body weight, 110 kg; body mass index, 35.5 kg/m^2^) and had a Glasgow Coma Scale score of 3 (eye-opening, 1; verbal response, 1; motor response, 1). His blood pressure was undetectable, pulse rate was 130 beats/min, and oxygen saturation was 96% on a 15 L/min reservoir mask. He was promptly intubated in the emergency department. Laboratory findings indicated liver dysfunction and hyperbilirubinemia (total bilirubin 2.8 mg/dL, aspartate aminotransferase 296 IU/L, alanine aminotransferase 138 IU/L), renal impairment (blood urea nitrogen 55.6 mg/dL, creatinine 2.80 mg/dL), an elevated inflammatory response (C-reactive protein [CRP] 19.94 mg/dL, white cell count 30.98 × 10^3/μL), and coagulopathy (prothrombin time international normalized ratio 1.60, activated partial thromboplastin time 42 s, d-dimer 80.6 μg/mL). The hemoglobin level (12.0 g/dL) and platelet counts (22.5 × 10^4/μL) were within relatively normal ranges. Contrast-enhanced computed tomography (CT) revealed an enlarged appendix, multiple bilobular ring-enhanced lesions in the liver, and a low-density area in the portal vein extending from the superior mesenteric vein (Fig. 1). Despite aggressive rehydration and noradrenaline administration (0.18 μg/kg/min) that improved the circulatory failure, the patient's hypoxemia, attributable to acute respiratory distress syndrome (ARDS), worsened. Consequently, venovenous extracorporeal membrane oxygenation (ECMO) was initiated in the emergency department. The patient was diagnosed with severe appendicitis, multiple liver abscesses, and portal vein thrombosis, all of which were complicated by septic shock and multiple organ failure. After intubation and mechanical ventilation, the patient was admitted to the intensive care unit, where antibiotic therapy with meropenem (MEPM) and anticoagulant therapy with heparin was initiated.

**Fig. 2:**
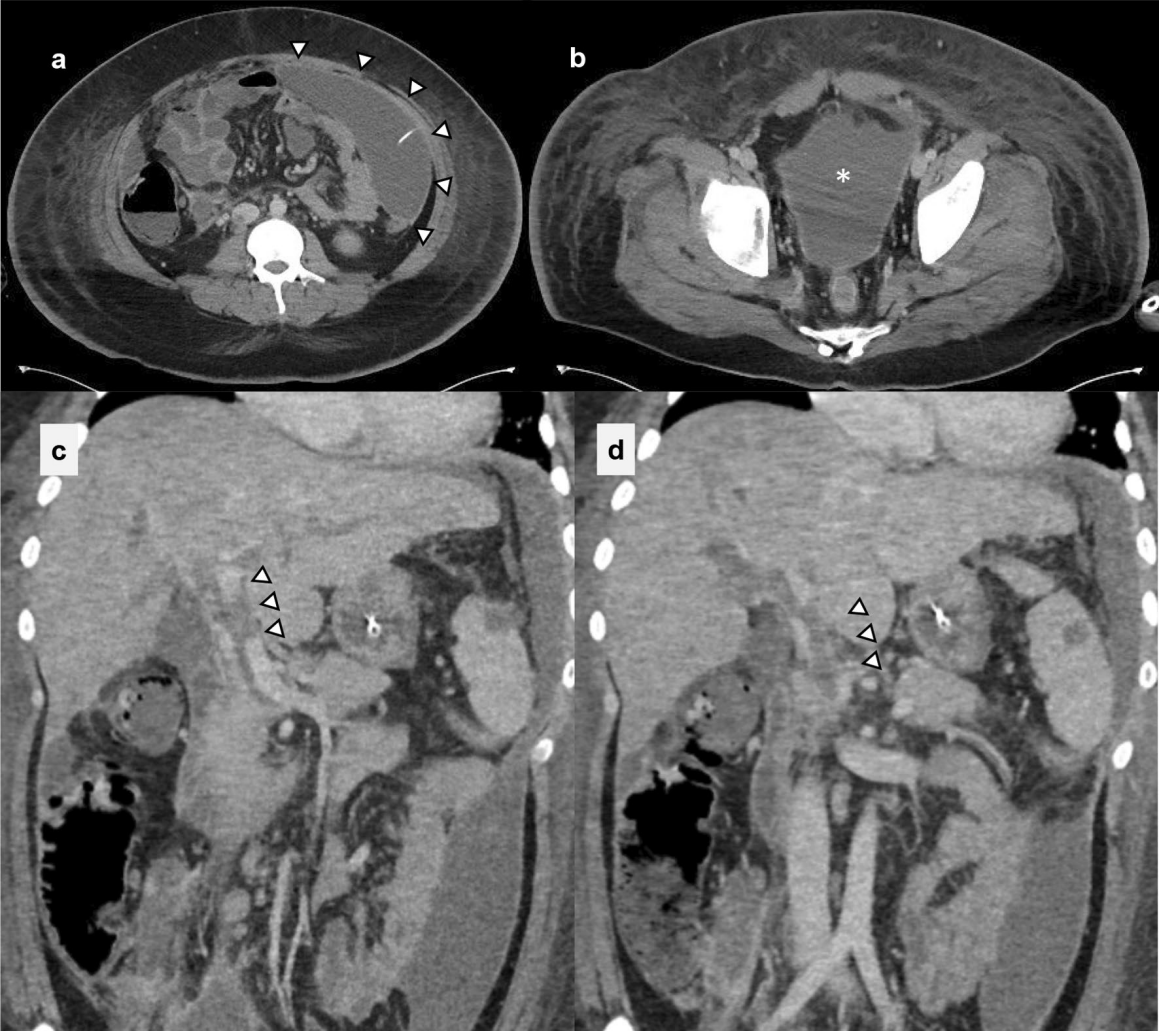
CT on day 10, when CRP levels started rising again, demonstrated **a** an abdominal paracentesis into an intra-abdominal abscess (arrowheads). **b** The abscess extended to Douglas's pouch (asterisk). **c** and **d** a low-density area in the portal vein of the sagittal sections (arrowheads). CT: computed tomography; CRP: C-reactive protein

**Fig. 3:**
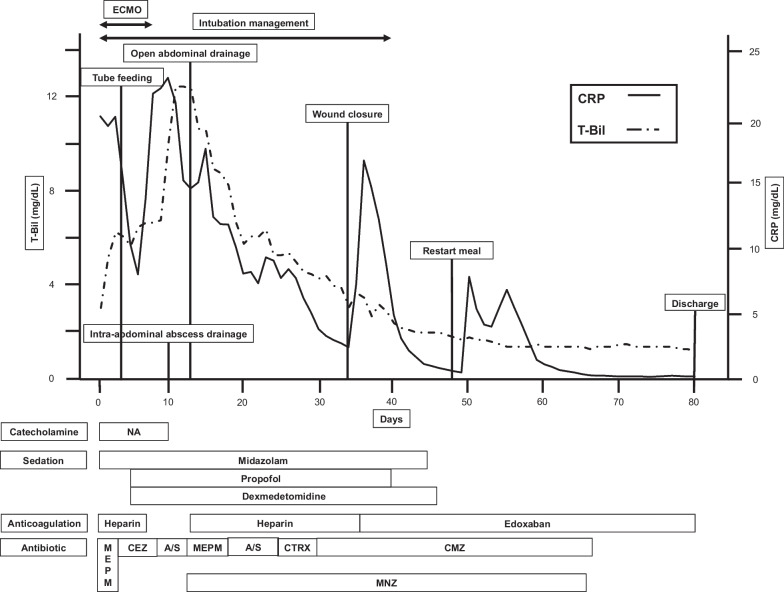
Trend in the CRP and T-Bil levels, drugs administered (catecholamine, sedation, and antibiotics), and medical events during the hospitalization. A/S: ampicillin/sulbactam; *B. fragilis*: *Bacteroides fragilis*; CEZ: cefazolin; CTRX: ceftriaxone; CMZ: cefmetazole; CRP: C-reactive protein; ECMO: extracorporeal membrane oxygenation; MEPM: meropenem; NA: Noradrenaline; T-Bil: total bilirubin

On day 6, the patient's respiratory condition improved sufficiently to facilitate weaning from the ECMO. However, by day 10, his CRP levels started rising again, and a subsequent CT scan demonstrated an intra-abdominal abscess extending from the inferior surface of the liver to Douglas's pouch, necessitating abdominal paracentesis. (Fig. 2). Cultures of both the ascitic fluid and blood revealed *Escherichia coli* (*E. coli*) and *Bacteroides fragilis* (*B. fragilis*). The strains were identified as carbapenemase (cfiA) gene-positive using matrix-assisted laser desorption/ionization time-of-flight (MALDI–TOF) mass spectrometry for protein spectrum analysis. They displayed resistance to a wide range of antimicrobials, including meropenem. Because the *B. fragilis* was susceptible to metronidazole (MNZ), this drug was added to the treatment regimen (Fig. 3).

**Fig. 4:**
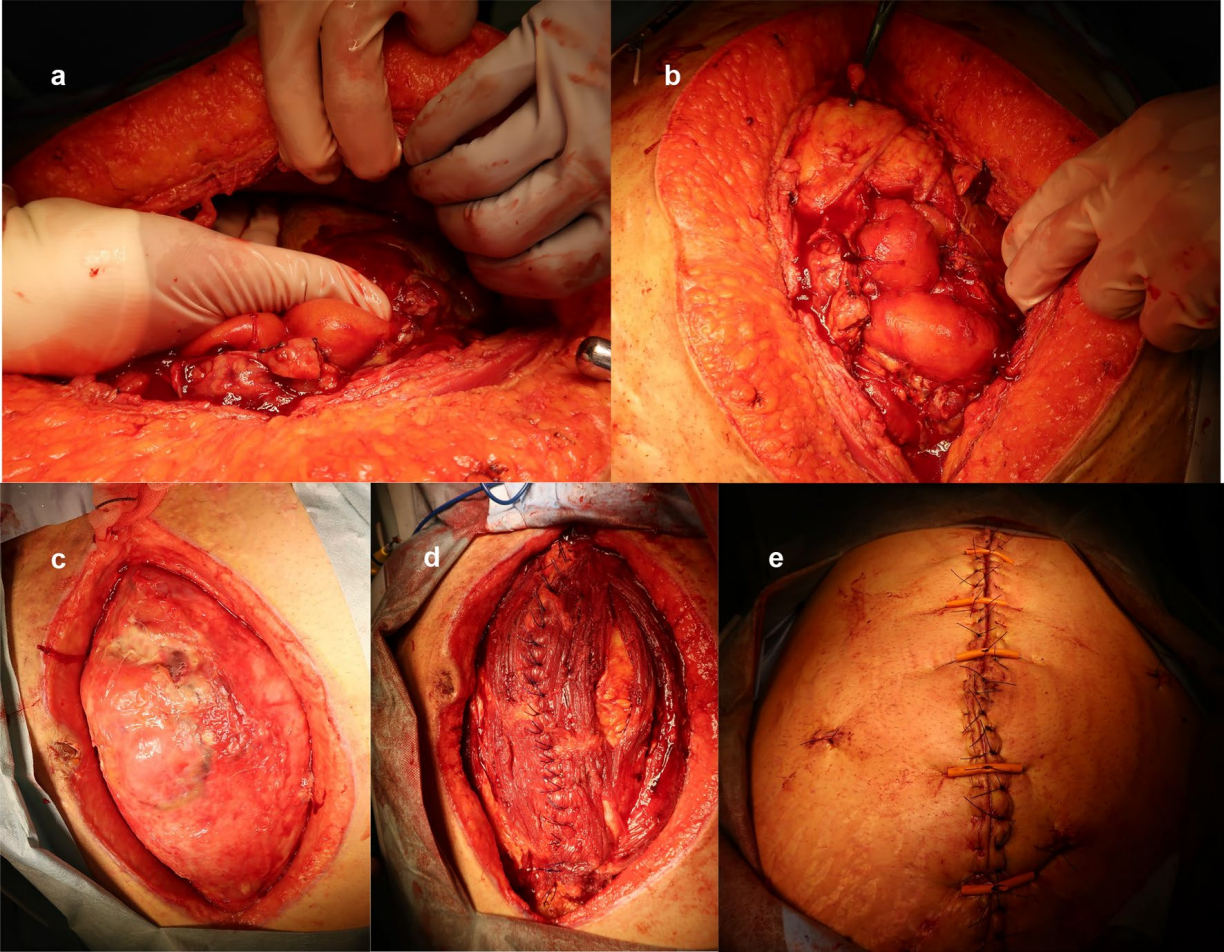
Intraoperative photographs. **a** On day 13, open abdominal cavity irrigation was performed. **b** During the laparotomy, the tissues were found to be highly hemorrhagic. **c**–**e** On day 34, the wound was closed using the anterior rectus abdominis sheath turnover technique with a relief incision

**Fig. 5:**
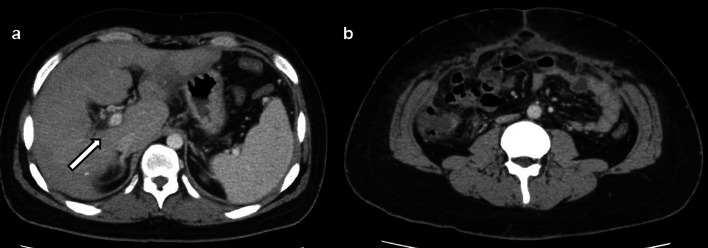
Abdominal CT image after discharge. CT findings demonstrated **a** the resolution of liver abscesses, portal vein thrombus (arrow), and **b** the intra-abdominal abscess. CT: computed tomography

Despite ongoing antimicrobial therapy and drainage procedures, laboratory data revealed persistently elevated CRP levels (Fig. 3). Therefore, on day 13, open abdominal cavity irrigation was performed for infection control. During laparotomy, the tissue was found to be extremely hemorrhagic owing to the continuation of the anticoagulant combined with the development of collateral blood vessels caused by portal vein thrombosis. After opening the intra-abdominal abscess, three drainage tubes were inserted into the bilateral paracolic gutters and Douglas's pouch (Fig. 4). The operation lasted 69 min with an intraoperative blood loss of 2711 mL, necessitating the transfusion of 8 units of fresh frozen plasma. Severe intestinal edema prevented wound closure, and the patient returned to the intensive care unit for OAM. Heparin administration was resumed on the following day. Intra-abdominal washout was performed every 5 days, and the patient's general condition gradually improved. Treating multidrug-resistant *B. fragilis* and *E. coli* involved using MNZ as the primary drug, supplemented with beta-lactamase inhibitors. However, prolonged antibiotic use led to hepatic dysfunction, but a change in the MNZ dose was not feasible. Accordingly, treatment was conducted using a strategy of suitably adjusting and co-administering beta-lactamase inhibitors. In the weekly follow-up blood cultures, a consistent absence of microbial growth was confirmed beginning from the third week of therapy. On day 34, the wound was closed using the anterior rectus abdominis sheath turnover technique with a relief incision. Treatment was successful, and the patient was discharged on day 81. A CT scan upon discharge confirmed the resolution of the intra-abdominal abscess, liver abscesses, and portal vein thrombus (Fig. 5).

## Discussion

Pylephlebitis, which is defined as infective thrombophlebitis of the portal vein, is relatively uncommon. The mortality remains notably high in the contemporary era despite advancements in antibiotic therapy and surgical interventions [1–3]. This condition typically manifests secondary to uncontrolled infections within regions drained by the portal and mesenteric venous systems [5]. Appendicitis and diverticulitis are the predominant causative factors [6–8]. Other underlying causes include cholecystitis, pancreatitis, malignancies, and infections localized within the abdominal and pelvic areas [9–13]. Specifically, in cases of appendicitis and diverticulitis, the combination of inflammation propagation from bacterial invasion, originating in the peripheral mesenteric veins and extending to the central portal vein, with a reduction in circulatory volume due to fever and dehydration, and an escalation in blood viscosity collectively contributes to septic thrombus formation within the portal vein [14]. The likelihood of developing septic portal vein thrombosis is significantly increased, particularly in cases of concomitant disseminated intravascular coagulation due to sepsis [15] (Fig. 5).

The diagnosis of pylephlebitis requires the confirmation of portal mesenteric vein thrombosis and bacteremia. Owing to the invasive nature of the procedures, proof of infectious portal thrombosis is seldom available. Thus, clinical presentation, radiographic findings, and cultures lead to the diagnosis [9, 16]. Clinical symptoms, including fatigue, fever, abdominal discomfort, nausea, vomiting, diarrhea, and anorexia, often manifest nonspecifically. Advanced symptoms include hepatomegaly and jaundice. Therefore, imaging studies that identify thromboses in the portal system are valuable diagnostic alternatives. Portal-phase imaging is favored in the acute phase not only because of its accessibility and the high-quality clinical data it provides but also because of its capability to discern complications, such as hepatic abscesses or intestinal ischemia [6, 17]. Nonetheless, early diagnosis can be elusive owing to nonspecific symptomatology. In this case, the condition was identified as more severe.

The therapeutic strategy for this condition involves three main steps: eradication of the causative pathogens, removal of infectious thrombi, and definitive management of the primary source of infection [16, 18]. However, there are no standardized treatment protocols. Current management approaches typically employ a combination of antibiotic therapy, surgical intervention, and anticoagulation, underscoring the need for prompt and appropriate intervention.

Antibiotic treatment consists of broad-spectrum antibiotics, with the choice of empiric antibiotics depending on the most probable source of infection and the most likely involved organisms, regardless of bacteriaemia [16]. When blood cultures yield positive results, treatments are tailored to target the identified pathogens. Blood cultures are advised for patients with suspected thrombotic portal vein phlebitis, as suggested in a previous study. However, blood cultures were positive at a previously reported rate of 42–62% [7, 9, 19].

Pylephlebitis is often a polymicrobial infection caused by gram-negative, gram-positive, aerobic, and anaerobic bacteria, especially *E. coli*, Streptococcus species Pluralis (spp.), and Bacteroides spp. According to previous reports, antibiotics were administered in almost all patients (94.6–100%) [7, 9, 19, 20]. In the present case, *B. fragilis* was isolated from both the blood and the intra-abdominal abscesses. *B. fragilis* is an obligate anaerobe that plays a pivotal role in the gut microbiota [21]. The recommended drugs for the empirical treatment of *B. fragilis* infections are active against aerobic bacteria, including fecal flora. Thus, the primary therapeutic choice is MNZ or piperacillin/tazobactam (PIPC/TAZ). Secondary options include carbapenems such as MEPM [22].

A noteworthy feature of this case was the multidrug-resistant nature of *B. fragilis*, which considerably impacted the antibiotic selection. Although resistance to MNZ, PIPC/TAZ, and carbapenems was once deemed exceedingly rare, recent global reports have suggested an increasing trend in the resistance of *B. fragilis* isolates to carbapenem antibiotics [23–26]. However, currently, in Japan, *B. fragilis* resistance to carbapenems is still rare (approximately 2%) [27–29]. In addition, carbapenem-resistant *B. fragilis* strains often exhibit an increased propensity to develop resistance to other anaerobic antibiotics, rendering them multidrug-resistant. The cfiA gene is specific to *B. fragilis*, encoding the carbapenem-hydrolyzing metallo-β-lactamase, and is expressed in 2–7% of the strains. The presence of the cfiA gene alone does not invariably confer carbapenem resistance; it has been postulated that an insertion sequence in the promoter region upstream of the cfiA gene might be responsible for pronounced carbapenem resistance [28, 30, 31]. In this case, the primary determinant for acquiring an infection caused by a multidrug-resistant strain is attributed to the patient's origin in China. In addition, using MALDI–TOF mass spectrometry for protein spectral analysis allowed for differentiation between cfiA gene-positive and gene-negative strains almost simultaneously with bacterial identification. Consequently, an antibiotic regimen centered on MNZ was promptly initiated.

Invasive therapeutic additions, such as the removal of the primary infection site or drainage of intra-abdominal abscesses via surgical or radiologically guided percutaneous approaches, are sometimes essential and effective in controlling the infection source. This case showed that management of the infection was challenging despite early identification of the causative bacteria, antibiotic susceptibility, and prompt antibiotic intervention. The appendectomy was deemed a critical component of the surgical treatment strategy. Nevertheless, owing to severe obesity and extensive involvement of abscess cavities during the procedure, the identification and excision of the appendix were deemed unfeasible. Therefore, we opted for surgical lavage drainage via laparotomy. OAM is also believed to yield the best outcomes. Generally, the objectives of OAM include prevention of increased intra-abdominal pressure, a straightforward approach to subsequent surgeries or lavages, and proliferation of abdominal infections [32, 33]. Using devices for provisional closure, OAM is sustained until control over bleeding, intra-abdominal contamination, and decreased intra-abdominal pressure was achieved, allowing for safe closure. The ability to effortlessly access and perform lavage of the abdominal cavity, even within the confines of an intensive care unit, has proven to be of immense value.

The role of anticoagulation therapy in the treatment of thrombophlebitis remains ambiguous owing to the infrequency of the condition and insufficient evidence. Thus, its implementation should be considered on a case basis. Situations warranting consideration for anticoagulant initiation include extensive thrombosis in the mesenteric vein, observed thrombus progression tendencies, persistent bacteremia despite antibiotic administration, or the presence of hypercoagulable states [20, 34]. This case demonstrated that anticoagulation therapy using heparin and edoxaban resulted in the resolution of the portal vein thrombus. After assessing the risk of hemorrhage and the potential worsening of portal vein thrombosis, we proceeded with surgical drainage, maintained anticoagulation therapy for portal vein thrombosis, and acknowledged the intrinsic bleeding risk. As a result, the diminution of the portal vein thrombus facilitates hemorrhage control from the collateral pathways, enabling secure abdominal closure.

## Conclusion

We reported a rare and successful case involving appropriate intensive care and the selection of antibiotics based on the susceptibility of the causative bacteria for septic shock and pylephlebitis arising from severe complicated appendicitis. Our findings underscored the value of early surgical drainage and open OAM when conservative measures, antibiotic therapy, and percutaneous drainage are challenging. In addition, our results emphasized the benefits of anticoagulation therapy to enhance the safety of surgical interventions.

## Data Availability

Data sharing is not applicable to this article, as no datasets were generated or analyzed during the current study.

## Abbreviations

SMV: Superior mesenteric veins
OAM: Open abdomen management
CRP: C-reactive protein
CT: Computed tomography
ARDS: Acute respiratory distress syndrome
ECMO: Extracorporeal membrane oxygenation
MEPM: Meropenem
*E. coli*: *Escherichia coli*
*B. fragilis*: *Bacteroides fragilis*
cfiA: Carbapenemase
MALDI–TOF: Matrix-assisted laser desorption/ionization-time of flight
MNZ: Metronidazole
spp.: Species pluralis
PIPC/TAZ: Piperacillin/tazobactam
